# The acute effects of boxing-specific dumbbell activity on punch performance in male amateur boxers

**DOI:** 10.3389/fphys.2025.1607933

**Published:** 2025-07-18

**Authors:** Rangxi Jin, Muyun Huang, Wenjuan Yi, Mitchell James Finlay, Chao Chen

**Affiliations:** ^1^ School of Athletic Performance, Shanghai University of Sport, Shanghai, China; ^2^ Sports Department, Wenzhou Cangnan County Lingxi Second Senior High School, Wenzhou, China; ^3^ Sports Department, Middle School Affiliated to Qingpu Teachers Training College of Shanghai, Shanghai, China; ^4^ Sport Department, University Academy 92, Manchester, United Kingdom; ^5^ Dalian University college of physical education, Dalian University, Dalian, China

**Keywords:** male amateur boxers, dumbbell throw (DBT), dumbbell push (DBP), fist velocity, post-activation performance enhancement (PAPE)

## Abstract

This study aimed to investigate and compare the acute effects of Dumbbell Throw (DBT) and Dumbbell Push (DBP) as punch-specific conditioning activities on subsequent punch performance in male amateur boxers, based on the post-activation performance enhancement (PAPE) framework. Eighteen participants completed maximal straight punch tests before and after conditioning activities (CA) performed with 2%, 5%, and 8% of 5RM bench press loads at 4, 8, 12, and 16 min post-CA. Punch velocity and power were measured using StrikeTec sensors. Significant CA × time interactions were observed for rear hand straight punches, with the 8% DBT condition producing peak velocity (9.81 m/s), power (29,824 W), and force (3,032 N) at 12 min post-CA. Compared to DBP, DBT led to greater improvements in rear fist velocity (+1.31 m/s, g = 1.57) and power (+6,154 W, g = 1.50). Jab performance peaked at 8 min post-CA with 5% DBT. Time main effects indicated overall enhancements. These findings suggest that DBT provides superior acute improvements in punch performance, likely due to its biomechanical specificity and stronger PAPE response. The optimal recovery time was identified as 8–12 min. Future research should include a control condition and further validate sensor-based measurements, while exploring optimal loading strategies.

## 1 Introduction

Boxing is a multi-directional, intermittent high-intensity combat sport, typically comprising between 2-4 rounds of 2–3-min duration, with a 1-min recovery between rounds (Davis et al., 2018). Previous research has shown straight punches to be important to success in boxing (Davis et al., 2015; Smith, 2010; Lenetsky et al., 2020). This punch, which is also known as the “cross” punch, comprises a kinetic sequence which starts with the production of ground reaction forces, rear leg drive and transfer of bodyweight from rear foot to front foot, rotation at the pelvis and trunk, and the propulsion of the upper extremities at high velocities prior to impact (Lenetsky et al., 2020; Lenetsky et al., 2013; Giovani and Nikolaidis, 2012; Smith et al., 2000; Cabral et al., 2010; Dyson et al., 2007). The rear hand straight punch comprises a greater trajectory, thus acceleration pathway when compared to another straight punch, the jab (Stanley et al., 2018). Unsurprisingly, the rear straight punch produces much greater force and distal velocities when compared to the jab. Indeed, previous authors have reported considerable impact forces in the rear hand straight technique (Finlay et al., 2024; Finlay, 2022; Dunn et al., 2022; Dunn et al., 2019; Loturco et al., 2016; Smith, 2006). Therefore, the development of an effective rear hand straight punch is desirable in delivering damage to an opponent via a knockout or in dominating a bout via an accumulation of strikes (Dunn et al., 2019). Experienced boxers can optimize their punching performance by integrating the dynamics of the entire body (Finlay et al., 2022). The proximal-to-distal sequencing—from the lower limbs through the trunk to the upper extremities—generates high velocities of the upper extremities. Indeed, leg drive is likely to influence pre-impact fist velocities (Lenetsky et al., 2013). Stanley et al., noted peak fist velocity of 6.97 (m/s) in the rear hand straight, with peak shoulder angular velocity of 534.5 ± 207.8 (deg/s), and peak elbow angular velocity of 399.6 ± 171.8 (deg/s) in the same punch. An effective punch may be one that yields large amounts of force, and/or achieves high distal-point velocities (Lenetsky et al., 2013; Giovani and Nikolaidis, 2012; Smith et al., 2000; Cabral et al., 2010; Dyson et al., 2007; Stanley et al., 2018; Finlay et al., 2024; Finlay, 2022; Dunn et al., 2022; Dunn et al., 2019), encompassing all of the kinetic sequencing described earlier. Whilst far from being the only factor in an effective punch (Lenetsky et al., 2013; Giovani and Nikolaidis, 2012; Smith et al., 2000; Cabral et al., 2010; Dyson et al., 2007; Stanley et al., 2018; Finlay et al., 2024; Finlay, 2022; Dunn et al., 2022; Dunn et al., 2019), the ability to propel the upper extremities at higher velocities is desirable for boxers. As such, the monitoring of kinematic variables such as velocity, in addition to punch force and power, may be interesting for coaches and practitioners working within boxing. In addition to strength and coordination, Wu et al. showed that visual reaction time and depth perception are also critical factors influencing punching performance in amateur boxers (Wu et al., 2024).

For practitioners and coaches, methods to acutely and chronically enhance performance in this technique, among other punches, becomes an important part of training. Regarding the former, a plethora of research has shown the potential for acute activity to enhance subsequent performance via a Post-activation Performance Enhancement (PAPE) effect (Terbalyan et al., 2025). PAPE is a phenomenon that manifests as an observable augmentation in neuromuscular output following intense voluntary muscular activities, termed conditioning activities (CAs) (Finlay et al., 2022; Terbalyan et al., 2025). The large amounts of research suggesting that a PAPE effect may emanate from prior voluntary muscular activity in several sporting and exercise tasks (Seitz and Haff, 2016), is mirrored in applied practice, where it is an extremely common practice in preparatory work to acutely enhance performance. More recently, this has been studied in combat sports too.

Based on this framework, selecting conditioning activities that closely replicate the biomechanics of the target movement is essential to maximizing the PAPE effect. In boxing, a widely used traditional practice involves punching with dumbbells—an approach believed to enhance fist velocity and power. Yet, scientific evidence supporting acute performance enhancement through dumbbell punching exercises remains limited (Lenetsky et al., 2013; Shirreffs and Maughan, 2006; Omcirk et al., 2023). This gap motivates the current study. Finlay et al. (2022) reported increases in the peak impact force of between ∼3 and 6% following a conditioning activity (CA) of either isometric punches, or punches with elastic resistance. Similarly, Yi al, et. reported increases in rear hand straight force and “velocity” following both ballistic and heavy loaded CA’s (Yi et al., 2022). Based on the aforementioned studies, there appears to be evidence of a PAPE effect on rear hand straight punch performance, emanating from upper-body ‘punch-specific’ CA’s, and also squat variations, lasting up to several minutes post CA. However, most existing studies on PAPE in boxing have focused on a single type of conditioning activity, such as isometric holds or elastic resistance, and often evaluated performance at only one or two time points post-activation. Furthermore, there has been limited exploration of load variation or biomechanical specificity within these protocols. Very few studies have directly compared different punch-specific CAs under controlled conditions. To our knowledge, no previous research has examined both push-based and throw-based dumbbell exercises in the context of PAPE, nor assessed their time-course and load-dependent effects on punch performance metrics. The current study therefore aims to fill this gap by systematically comparing the acute effects of dumbbell push (DBP) and dumbbell throw (DBT) across three load intensities and four time intervals, providing a more comprehensive understanding of how punch-specific CAs modulate performance. This approach allows us to identify both the most effective modality and optimal timing for practical application in boxing training.

Despite its prevalence, this practice has drawn criticism, as the natural deceleration during the punch may reduce specificity and potentially hinder transfer to actual performance (Lenetsky et al., 2013). Although landmine punches and presses are commonly incorporated into strength and conditioning routines, their relevance to actual punching mechanics—particularly in terms of high-velocity release—has primarily been questioned in practitioner circles, with limited empirical research available to support or refute these claims (Turner, 2009). Both exercises include logistical considerations of course. Nevertheless, whilst the longer-term training benefits of “punching with dumbbells” has not been studied in great detail, this is also true of the ability of the above movements to induce more acute effects on punch-specific performance. Further questions on whether this traditional boxing training modality can acutely enhance performance, lies within the load of the dumbbells and repetitions performed. Knowledge of how effective punching with dumbbells may be in inducing PAPE in punch-specific performance, may be useful to coaches and practitioners managing the acute preparatory work of the boxing athlete.

Therefore, this study focuses on the potential PAPE inducing effects of the punch-specific dumbbell push (DBP), and the dumbbell throw (DBT) on subsequent straight punch performance. In addition, this study aims to identify the key factors influencing any PAPE effect, such as load volume, recovery time, and their specific impact on boxers’ performance (Zhou et al., 2022; Kamali et al., 2021; López-Laval et al., 2020). Based on prior literature, it is hypothesised that a greater pre-post-performance benefit will be observed following the dumbbell throw (DBT) condition compared to the dumbbell push (DBP) condition. This hypothesis is grounded in previous findings showing that conditioning activities involving ballistic or release-based actions—such as medicine ball throws or bench press throws—are more effective in eliciting PAPE effects than non-release movements, likely due to enhanced movement intent, reduced deceleration forces, and higher neural activation (Yi et al., 2022; Finlay et al., 2022; Judge et al., 2010). Moreover, the dynamic nature and release mechanics of the DBT more closely replicate the proximal-to-distal sequencing found in actual punching techniques, thereby offering superior biomechanical specificity.

## 2 Materials and methods

### 2.1 Experimental approach to the problem

A within-subject repeated-measures cross-over design was used to assess the influence of two punch-specific CAs on subsequent straight punch performance. Boxers were asked to conform to pre-testing controls, which included the avoidance of vigorous activity and consumption of alcohol or stimulants for 48-h prior to each testing session (Shirreffs and Maughan, 2006). During the experiment, participants were asked to wear standard athletic clothing and their usual boxing shoes to eliminate the potential influence of different clothing on the results of the experiment. Likewise, during the punching trials, participants wore their usual hand wraps and gloves used in sparring. The two CAs were the dumbbell push, and the dumbbell throw. Prior to the dumbbell push or the dumbbell throw, participants performed a standardized warm-up. Details of the CA’s and the standardised warm-up can be found later in the method section. The experimental design included two independent variables: dumbbell weight and activity type. Dumbbell loads in both conditions were set at 2% 5RM, 5% 5RM and 8% 5RM in the bench press (discussed later in the section). Likewise, in both trials, punch performance tests were carried out at 4, 8, 12, and 16-min post CA. The specific loads—2%, 5%, and 8% of the participant’s 5RM—were selected based on prior evidence suggesting that loads exceeding ∼10% of an individual’s maximal strength or body weight can reduce movement velocity, disrupt technique, or impair subsequent performance in explosive and ballistic tasks (Kamali et al., 2021; Omcirk et al., 2023; Shirreffs and Maughan, 2006). By selecting a low-to-moderate load range, we aimed to balance sufficient neuromuscular stimulus for PAPE without inducing excessive fatigue or compromising punching mechanics. These values also allowed us to explore potential dose-response effects across a realistic intensity spectrum for applied training environments.

### 2.2 Subjects

Eighteen trained boxers from the Shanghai University and affiliated competitive schools were selected as participants for this study. All boxers were currently completing a minimum of 14 h of boxing training per week. All participants were made aware of the study aims, the benefits and potential risks, and asked to provide informed consent. Verbally confirmation that they were free of injury at the time of testing, was also required. The research procedure has been approved by the Ethics Committee of Shanghai University of Sport (Ethics number:102772021RT031) and is in accordance with the latest version of the Declaration of Helsinki (Association 2013). All participants signed a written notification form. All participants had similar training backgrounds, with a minimum of 14 h of weekly boxing training, and were recruited from comparable competitive environments to reduce training-level variability. However, potential individual differences in anthropometrics and experience were not used as covariates in statistical analysis. All participants attended a familiarization session 48 h prior to data collection, during which they practiced both conditioning activities (DBT and DBP) and performed punches using the StrikeTec sensor setup. This session ensured procedural familiarity and minimized learning effects during formal testing. All participants were trained amateur boxers competing at the collegiate or regional level. They had a minimum of 2 years of structured strength and conditioning (S&C) experience, including regular exposure to resistance training exercises such as the bench press. All participants were familiar with the conditioning exercises used in the study (DBT and DBP), having incorporated similar punch-specific drills in their weekly training.

## 3 Procedures

### 3.1 5RM bench press

Participants performed their 5RM bench press in order to calculate dumbbell load in the CA’s. Initially, participants performed a brief warm-up of low load bench press repetitions, followed by performs a light load warm-up, followed by a 1-min rest. Thereafter, the load is gradually increased. If the participant can complete 7–10 repetitions, then they rest for 2 min before attempting the 5RM bench press, which involves completing 5 repetitions. If successful, then they rest for 2–4 min before increasing the load by 2 kg . If unsuccessful, then the load is reduced by 2 kg after a rest period until the athlete can complete 5 repetitions with proper bench press technique. This 5RM protocol follows established guidelines for submaximal strength testing in trained populations (Baechle and Earle, 2008). The participant adhered to the correct bench press technique instructed and demonstrated by the lead researcher prior to the test and completed this under the supervision of the lead researcher. Participants would perform the CA’s disat 2, 5, and 8% of the 5RM obtained in the above test.

### 3.2 Conditioning activities (CA’s)

All experimental sessions were conducted in the same indoor training facility under controlled temperature (21°C–23°C) and lighting conditions. Each participant was tested at the same time of day (±1 h) across sessions to minimize circadian-related performance variation. Standardized verbal instructions were provided by the same researcher throughout all trials. Prior to both trials, participants completed a RAMP warm-up consisting of 10 min of jogging and dynamic stretching, followed by 5 minutes of boxing specific activity. A baseline performance test was then performed following the warm-up, followed either by the dumbbell throw (DBT) or dumbbell push (DBP), The brand of dumbbell is Technogym, the model and specifications are the same. discussed below. To control order and fatigue effects in this repeated-measures crossover design, participants were randomly assigned to begin with either the DBP or DBT protocol using a computer-generated randomization list. The order of conditions (DBP/DBT) was counterbalanced across participants. Each dumbbell load condition (2%, 5%, and 8% of the participants’ 5RM) was tested on a separate day to avoid cumulative fatigue. Load order was randomized and counterbalanced across testing days. Within each session, participants performed 6 repetitions per arm (lead and rear hand), and the order of arms was randomized using a computer-generated sequence.

### 3.3 Dumbbell throw (DBT)

In the DBT condition, the participants performed 1 set of 6 repetitions in the lead and rear hand, with each repetition interspersed with 10s recovery. For each load condition, participants performed 6 repetitions with the lead hand followed by 6 repetitions with the rear hand, totaling 12 repetitions per load. All repetitions were performed from a standing position, with participants in their orthodox boxing stance, to simulate real punching mechanics. As mentioned above, this DBT condition served as a “release” condition, whereby participants released the DBs once near full elbow extension. Prior to testing, participants were given standardized verbal instruction and a live demonstration by the lead researcher to ensure uniform technique. The DBT was intended to replicate the explosive, ballistic characteristics of a straight punch, including proximal-to-distal force transmission and maximal intent. All repetitions were supervised to ensure consistency across trials. Participants maintained the non-active hand in a defensive guard position (close to their chin and face) during each repetition, mimicking realistic boxing mechanics. Punching direction was standardized as a horizontal forward motion aimed at shoulder height. Participants were explicitly instructed to use controlled trunk rotation to maximize biomechanical specificity. The dumbbell was gripped firmly around the handle, with participants stabilizing their wrists and ensuring a neutral wrist alignment throughout each throw.

### 3.4 Dumbbell push (DBP)

Similar to the DBT CA, in the DBP condition, the participants performed 1 set of 6 repetitions in the lead and rear hand, with each repetition interspersed with 10s recovery. For each load, participants completed 6 repetitions per arm (lead and rear), with the order of arms randomized. These were also performed in a standing position in orthodox stance, maintaining biomechanical similarity to boxing punches. Although the dumbbell was not released, participants were instructed to extend the arm rapidly and then decelerate under control to simulate a punch with stopping intent, as often performed during technical drills. Movement intent and joint sequencing were emphasized to approximate real striking mechanics. The DBP essentially served as a “non-release” condition, whereby the DBs remained in the participants hands throughout the movement. Like the DBT protocol, participants kept the non-active hand in a defensive guard position (near their chin and face) throughout the DBP repetitions. Punching direction was standardized as a horizontal forward motion aimed at shoulder height. Trunk rotation was again emphasized and explicitly instructed to participants. Participants maintained a firm grip on the dumbbell handle, stabilizing their wrists and keeping a neutral alignment throughout the movement, ensuring controlled deceleration at full extension.

### 3.5 Punch performance

Participants punch performance in maximal straight punches was assessed via the commercially available StrikeTec boxing-specific accelerometers (StrikeTec Boxing Sensors, StrikeTec, Dallas, TX, USA, v1.4.4). There is limited literature on the above devices; however, one study (Bliss et al., 2021) reported that when the StrikeTec technology was successful in registering punches, users can accept the velocity data, due to moderate correlations with QMV (Qualysis–mean velocity) ( r = 0.56, mean absolute percentage error [MAPE] = 1.49, mean percentage error [MPE] = 1.49) and QPV (peak velocity) ( r = 0.55, MAPE = 0.46, MPE = 0.43). Power and Force variables from the same study performed less well, and as such, no further analyses on the aforementioned variables were conducted. Nevertheless, in the present study, these metrics were recorded and reported to reflect practical usage trends, as they are standard outputs provided by the sensor and often utilized in applied sport environments. While we acknowledge their limited validation, their inclusion was aimed at exploring potential differences between conditions. We urge readers to interpret these data with caution and emphasize the need for future studies to validate these metrics aegainst laboratory-based measures.

As mentioned, the participants wore their own competition hand wraps and gloves. The StrikeTec devices were placed at the wrist extensors on top of the hand wraps, held tightly in place with a further 2.5 m of wrap as per Omcirk et al., (Bliss et al., 2021). Finally, 12 oz boxing gloves were applied. The participants adopted their boxing stance, in which all boxers in the study were of orthodox stance, and awaited instruction from the lead researcher. Participants were instructed to perform each punch with maximal effort and velocity, simulating the intent and intensity of a real competitive punch.

Upon hearing the instruction, the participants performed 3 jabs, followed by 3 rear hand straights against a human shaped sandbag (Jiurishan, China). The interval between each punch was 15 s, and participants were advised to remain in their set stance, thus same distance away from the target. To ensure consistency in distance, a fixed floor marker was used to indicate the optimal position for each participant’s lead foot. All punches were performed from this mark, which was aligned at a standardized distance from the dummy bag. This setup was maintained across all trials and conditions. Mean velocity, and “power” was recorded in the StrikeTec mobile application, and was manually inputted to a Microsoft Excel spreadsheet. In this study, punch performance was assessed using three key metrics: velocity (m/s), power (W), and force (N). These represent the speed of the striking hand, the rate of force production, and the impact magnitude, respectively.

### 3.6 Statistical analysis

Following confirmation of the normality of data, a repeated measures ANOVA (3x5) was performed to determine the effects of CA type and time on punch performance. Where significant main effects and interactions were identified, *post hoc* pairwise comparisons with a Bonferroni correction were applied. All analyses were performed via SPSS v.22.0 (IBM Inc., 277 Chicago, IL), with statistical significance assumed at P ≤ 0.05. All data are reported as mean ± SD, %, or effect size (Hedges g ) unless otherwise stated. Effect sizes were calculated using Hedges’ g, which accounts for small sample bias. Following sport science-specific guidelines (Rhea, 2004; Hopkins et al., 2009), effect sizes were interpreted as small (g = 0.25–0.49), moderate (0.50–0.99), and large (≥1.00) for trained individuals. This framework provides a more meaningful context for understanding the practical significance of performance changes in this athletic population (Rhea, 2004; Hopkins et al., 2009).

## 4 Results

### 4.1 Participants characteristics

Participant age and anthropometric data were as follows; age 23.6 ± 2 yrs ; boxing training experience 7.2 ± 1.6 yrs ; height 175 ± 3 cm ; mass 65.0 ± 6.3 kg ; bench press 5RM 73.7 ± 2.7 kg .

### 4.2 Fist velocity

A 2-way repeated measures ANOVA revealed a significant CA × time interaction for rear hand straight velocity (P = 0.028, η^2^p = 0.243). There was no significant main effect for CA (P = 0.181, η^2^p = 0.089) or time (P = 0.097, η^2^p = 0.131). Post hoc analysis indicated that at 12 min post-CA, fist velocity in the 8% DBT condition was significantly higher than in the 8% DBP condition (mean difference = +1.31 m/s, 95% CI [0.61–2.02], P = 0.041 (Bonferroni corrected), g = 1.07 [0.62–1.65]). Though no significant interaction was observed for jab velocity (P = 0.221, η^2^p = 0.115), a main effect for time was found (P = 0.048, η^2^p = 0.198).

**FIGURE 1:**
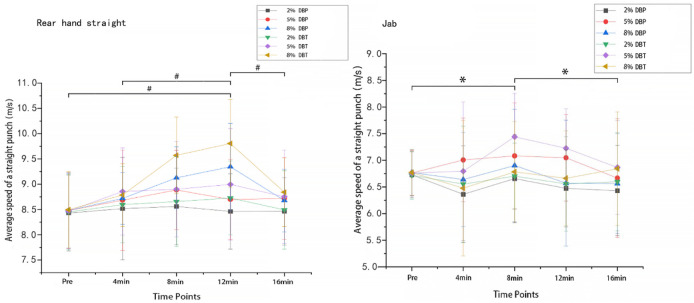
Average Velocity of Straight Punches on the rear hand straight and jab hand straight (units: m/s, W, N). Note. # indicates that punching velocity in the 8% DBT training group on the rear straight at the 12-min time point was significantly different from those at pre-, 4 min, and 16 min * indicates that punching velocity in the 5% DBT training group on the jab straight at the 8-min time point was significantly different from those at pre- and 16 min.

In contrast to the rear hand straight, there was no significant interaction between CA and time for average velocity in the jab, and also no main effect for CA. Figure 1b highlights the significant main effects of time ( p < 0.05). The largest increase in pre-post fist velocity in the rear hand straight was observed in the 8%DBT trial at 12-mins post-CA (+1.31 m/s, [Hedges] g = 1.57). At 12 min post-CA, the rear hand straight velocity was also significantly higher in the 8% DBT condition compared to the 8% DBP condition (Hedges’ g = 1.07), representing a large effect size and suggesting a meaningful performance benefit. Although these differences should be interpreted cautiously given the device limitations, a +1.31 m/s increase in fist velocity may still represent a meaningful advantage in a sport where marginal gains can affect scoring and tactical success. The corresponding large effect size (g = 1.57) further suggests that the change is not only statistically significant but potentially relevant to performance. At the same time point, the corresponding 8%DBP trial showed pre-post increases of +0.87 m/s ( g = 1.06). For the jab, the greatest pre-post change was seen in the 5% DBT trial at 8-mins post-CA (+0.68 m/s, g = 1.02), whilst at the same time point, the corresponding 5% DBP trial induced pre-post changes of +0.32 m/s ( g = 0.41).

### 4.3 Punch power

A 2-way repeated measures ANOVA revealed a significant CA × time interaction for rear hand straight punch power (P = 0.017, η^2^p = 0.312). There was no main effect for CA (P = 0.109, η^2^p = 0.156) or time (P = 0.083, η^2^p = 0.143). Post hoc comparisons indicated that at 12 min post-CA, the 8% DBT condition resulted in significantly greater punch power than the 8% DBP condition (mean difference = +2130.5 W, 95% CI [1088.4–3172.6], P(Bonf) = 0.037, g = 1.28 [0.71–1.84]). For the jab, no significant CA × time interaction was observed (P = 0.236, η^2^p = 0.107), though a main effect of time was found (P = 0.046, η^2^p = 0.201). The 5% DBT at 8-min post-CA showed the highest improvement in jab power (+3533.5 W, g = 1.16, 95% CI [1984.7–5082.2]).

**FIGURE 2:**
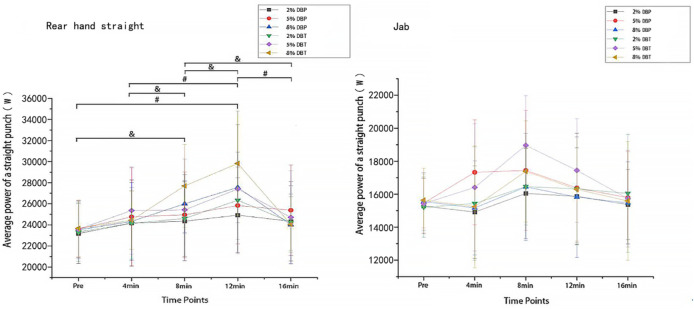
Average Power of Straight Punches on the rear hand straight and jab hand straight (units: m/s, W, N). Note. & indicates that punching power in the 8% DBT training group on the rear straight at 8 min was significantly different from those at pre-, 4 min, 12 min, and 16 min # indicates that punching power in the 8% DBT training group at 12 min was significantly different from those at pre-, 4 min, 8 min, and 16 min.

In the jab, there was no significant CA × time interaction (p = 0.236, η^2^_p_ = 0.107), and no significant main effect for CA (p = 0.391, η^2^_p_ = 0.084). Figure 2b shows the significant main effects of time ( p < 0.05). For the jab, the greatest pre-post change in punch power was seen in the 5% DBT trial at 8-mins post-CA (+3533.5 W , g = 1.16), whilst at the same time point, the corresponding 5% DBP trial induced pre-post changes of +1979.8 W , g = 0.69.

### 4.4 Punch force

**FIGURE 3:**
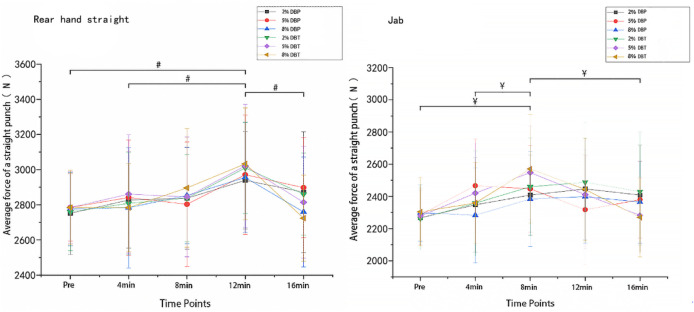
Average Force of Straight Punches on the rear hand straight and jab hand straight (units: m/s, W, N). Note. # indicates that punching force in the 8% DBT training group on the rear straight at 12 min was significantly different from those at pre-, 4 min, and 16 min ¥ indicates that punching force in the 8% DBT training group on the jab straight at 8 min was significantly different from that at pre-, 4 min, and 16 min.

A 2-way repeated measures ANOVA showed no significant CA × time interaction for rear hand straight punch force (P = 0.272, η^2^p = 0.093). There was a main effect of time (P = 0.022, η^2^p = 0.218), but not for CA (P = 0.129, η^2^p = 0.121). Post hoc testing revealed that punch force peaked at 12 min post-CA in the 8% DBT condition (mean = 3032 ± 318 N), significantly higher than pre-CA (mean difference = +418.7 N, 95% CI [161.1–676.3], P(Bonf) = 0.029, g = 1.14 [0.53–1.66]). For the jab, the 8% DBT condition at 8 min post-CA showed significantly higher force than pre-CA (P = 0.042, η^2^p = 0.191, g = 0.87 [0.34–1.37]). As shown in Figure 3.

## 5 Discussion

### 5.1 Overall findings and key performance effects

This study aimed to compare the effects of dumbbell push (DBP) and dumbbell throw (DBT) conditioning activities (CA), on punch-specific performance in amateur boxers. In accord with the studies hypothesis, greater performance increases in the rear hand straight, were observed following the CA that incorporated a throw (DBT). A general trend of increased rear hand straight punch performance, observed in the available metrics from the devices (velocity, power, force) was evident following each CA and load. The acute performance enhancement of velocity, power, and force was greatest at 12-min following the 8%5RM DBT condition, with values of 9.81 ± 0.87 m/s, 29,823.9 ± 4,967 W, and 3,032 ± 318 N found, respectively. The above values and reported effect sizes represent the greatest punch performance increase observed in the whole study. Finlay et al. similarly reported that upper-body isometric and elastic resistance CAs produced a 5%–7% increase in punch-specific neuromuscular performance in amateur boxers, reinforcing the applicability of targeted upper-body activation (Finlay et al., 2024). Importantly, even these relatively modest absolute gains can have meaningful implications in competitive contexts. From a practical perspective, even modest increases in fist velocity (e.g., +1.31 m/s) and power (e.g., +6154 W) can meaningfully influence competitive outcomes in amateur boxing, where rapid punch execution and forceful impact play crucial roles in scoring, maintaining offensive pressure, and controlling bout rhythm. These findings suggest that DBT-based conditioning protocols may be a valuable addition to pre-bout warm-up strategies. Similarly, performance in the jab improved following select CA’s and loads, though this acute enhancement was more apparent following the 5%5RM DBT condition, at 8-min. Overall, the findings suggest that a DBT CA included in a warm-up, may induce greater performance increases in fist velocity and power of the rear hand straight and jab punches, when compared to a DBP CA. The findings suggest consideration to different loads and recovery times is required, with the latter found to be optimal between 8 and 12-min post-CA, in partial agreement with previous research.

### 5.2 Punch type differences and temporal response

In contrast to the jab, the rear hand straight punch is primarily used to inflict damage on an opponent, thus for it to be effective, it must be delivered with substantial force, and typically at high velocities. In the present study, velocity and power peaked at 12-mins post both DBT and DBP CA’s, whilst these variables were also heightened at the 8-min recovery interval, compared to baseline. This is in line with prior research that suggests an acute increase in performance may be seen for several minutes post-CA (Terbalyan et al., 2025; Blazevich and Babault, 2019; Blazevich and Babault, 2019; Seitz and Haff, 2016; Yi et al., 2022; Lenetsky et al., 2013; Turner, 2009; Zhou et al., 2022). Performance in that study was also heightened compared to baseline at the 11 and 13th minute mark. It is worth noting, however; that whilst the CA’s shared similarities with a punching action, one was performed as a maximal voluntary contraction (MVC), and the other with elasticated resistance. Further, the aforementioned study assessed punch impact force directly via a vertically mounted force plate. Yi et al., also reported peak improvements in rear hand straight performance at 9-min post a ballistic CA (Yi et al., 2022). These findings align with those of Finlay et al., who observed improved punch force after isometric and resistance-band-based CAs. However, our study expands upon this by introducing a comparison between two movement-specific, load-bearing conditioning activities. Similarly, while Yi et al. focused on ballistic lower-body movements (e.g., squats), our data provide novel evidence for upper-body punch-specific CAs that vary by release mechanics and load intensity. A plethora of research (López-Laval et al., 2020; Finlay et al., 2022) suggests that were added to an appropriate warm-up, a CA that shares biomechanical specificity to the performance task, may induce acute improvements in said task. The proximal-to-distal sequencing observed in a punching action, particularly in the rear hand straight discussed earlier, is also evident in the two CA’s explored in the current study. This specificity, whether intentional or not, and whether applied correctly or not, may be the reason why ‘punching with dumbbells’ is a traditionally used training modality in boxing. Unlike earlier studies that typically used a single CA type or limited observation time points, our study incorporated both push-based and throw-based upper-body CAs across three load levels and four post-activation time intervals. This comprehensive design enables a deeper understanding of how exercise modality, intensity, and timing interact to influence punch performance, providing more actionable insights for performance practitioners.

### 5.3 The jab and its tactical importance

The jab also saw improvements in fist velocity and power. Whilst it is not a damaging punch, it is desirable for boxers to have a rapid jab for increased effectiveness to manage distance, and to find range for a subsequent punch. As such, the increased velocity and power of the punch emanating from the DBT CA is promising. Both CAs seemingly induced a performance increase in straight punch performance, and one reason may be due to the increased intent within the punching action.

### 5.4 PAPE mechanisms and movement intent

The specific mechanisms that explain PAPE is still lacking, thus interpretation of this studies results comprise some speculative commentary. Although we hypothesized that greater neural activation and movement intent may explain the superior performance gains in the DBT condition, these remain speculative interpretations. The current study did not include electromyography (EMG) or other neuromuscular assessments to directly verify differences in muscle activation or coordination between conditions. Future research should incorporate EMG to validate the proposed mechanisms and provide more definitive insight into how different conditioning activities modulate punch-specific performance. However, recent work has described this as shared mechanisms that we observe from a general warm-up (i.e., increased muscle activation and temperature-related mechanisms), leading to some academics and practitioners to question whether the PAPE effect is merely just a warm-up effect. Irrespective of the above, there is potential for acute performance increases emanating from purposefully designed CA’s, to be of a greater magnitude than that produced by the preceding warm-up alone (Wu et al., 2024). The addition of a CA and manipulation of the intensity, repetitions, and recovery period may alter the PAPE-fatigue balance (Seitz and Haff, 2016).

### 5.5 DBT, biomechanical specificity, and literature context

While prior literature shows mixed results with overloaded implements, the superior effect observed in the DBT condition may be attributed to its biomechanical compatibility with the punching motion and its capacity to promote neuromuscular activation through release mechanics. One plausible explanation for the greater effect of DBT is its alignment with the natural force-generation sequence in punching—starting from the lower limbs, transferring through the hips and torso, and culminating in the arm and fist. The DBT condition allows for full elbow extension and release, minimizing the need for deceleration and enabling a complete expression of force through the kinetic chain. This release component may also increase movement intent and neural activation, both of which are key contributors to effective post-activation performance enhancement. In contrast, DBP constrains the terminal phase of the punch due to the need to decelerate the dumbbell internally, potentially disrupting force flow and reducing specificity to real boxing mechanics.

### 5.6 Potential conflicts in literature on weighted implements

Prior research is conflicted on the effectiveness of “overweighted” implements in inducing an acute performance benefit in the same, or a similar task under ‘normal’ conditions. For example, in literature on throwing sports, the use of “overweighted” instruments may acutely enhance subsequent performance in throwing and performance compared to a control trial (Judge et al., 2010; Bellar et al., 2012). In contrast, a study exploring the use of cricket bowling with a 10% heavier cricket ball, found it did not induce an acute performance benefit on subsequent bowling accuracy and velocity compared to a control trial (Feros et al., 2021). The dumbbells used in the present study may be considered “weighted implements” of sorts. The literature is seemingly more conflicted when it comes to weighted implements in hitting sports. Bliss and colleagues reported an increase in clubhead velocity in a golf swing following a warm-up that included overweight implement swings, compared to a control trial (Bliss et al., 2021). However, (and pertinent to the discussion of the viability and practicality of ‘sport-specific’ CA’s), when comparing the weighted implement trial to one of brief general bodyweight activity, no significant differences were found. In a separate study, a similar CA showed potential in improving club head velocity by up to 2.4 mph and increases in peak angular velocity of the upper extremity locations of interest, and the club itself (Hébert-Losier and Wardell, 2024). Three studies have shown a lack of improvement, and in some cases a detrimental effect on baseball swing performance, when prior swings are performed with a heavier bat (Montoya et al., 2009; Williams et al., 2019; Higuchi et al., 2013).

### 5.7 Movement intent and load specificity

The use of ‘weighted’ activity could contribute to the development of motor patterns at lower velocities (Judge et al., 2010), and this potential interference with punching technique via punching with dumbbells in the context of the present study, may explain why some academics and practitioners in combat sports avoid this traditionally used training modality. However, the current study explored it’s use as an acute stimulus only. The positive results emanating from the CA’s may be explained by the increased intent, defined as the athlete’s conscious effort to move with maximal speed and force, due to the intensity of the action due to its intensity, and its effect on muscle recruitment and neural activation. Specifically, boxers were asked to perform the action as rapidly as possible, and this intensity, particularly in the throw condition, may have had an increased impact on muscle recruitment and neural activation. This may explain why the greater loads of 5 or 8%5RM were seemingly more effective. However, it is worth noting that muscle recruitment and neural activation was not quantified in the present study, and as such, is speculative.

### 5.8 PAPE vs. fatigue: Recovery timing matters

Another important factor in PAPE research, and one already explored briefly in this section, is the dynamic between intensity and recovery, on the balance between performance enhancement (or historically termed potentiation) and fatigue. Judge, Bellar, and Judge noted the potential for an overweighted activity to fatigue athletes for subsequent performance, potentially explaining why an implement that was 1.37 kg overweight, enhanced subsequent throwing performance compared to a control, whereby an implement that was 2.27 kg overweight, did not. This co-existence and balance of PAPE and fatigue, dependent on implement load and recovery time, can be seen in the results of the present study (Judge et al., 2010). For example, at 4-min there was generally a lack of positive improvements noted. In theory, this could suggest that the balance between fatigue and performance enhancement was tipped in the favour of the former, or that performance benefits had not yet manifested. Between 8 and 12-min was the optimal recovery required for performance enhancement to occur, and fatigue to subside, in keeping with the range found in PAPE literature within boxing as mentioned earlier (Blazevich and Babault, 2019; Blazevich and Babault, 2019; Seitz and Haff, 2016; Yi et al., 2022; Shirreffs and Maughan, 2006; Omcirk et al., 2023; Finlay et al., 2022).

### 5.9 Limitations and recommendations for future research

This study had several limitations that should be acknowledged. First, the absence of a “no-CA” or warm-up-only control group limits the ability to isolate the specific effects of the conditioning activities from the baseline warm-up. This weakens the strength of causal inference and makes it difficult to determine how much of the observed performance enhancement was due to the intervention itself. Future studies should include a true control condition to better distinguish CA-induced effects from warm-up responses. Second, although the StrikeTec sensors provided convenient punch-specific metrics, their validity—especially for force and power measurements—has not been conclusively established. This introduces potential measurement error and may explain some of the variability in results. It is critical for future research to validate such wearable sensors against gold-standard laboratory equipment. Third, while participant characteristics (e.g., height, weight, training experience) were recorded, no statistical analysis was conducted to examine how these individual differences influenced the PAPE response. This limits our ability to identify which types of athletes may benefit most from specific CAs. Future studies should explore this via stratified or regression analysis. Collectively, these limitations suggest the need for further validation of measurement tools, inclusion of appropriate control conditions, and individualized analysis to better understand the complex interactions between conditioning variables and performance outcomes. In addition, although punch force and power data were reported using StrikeTec sensors, we acknowledge that these metrics lack full validation, unlike velocity data which showed moderate correlations in prior studies (Omcirk et al., 2023). As such, the interpretation of force and power results in the current study should be made cautiously, and future research is encouraged to further validate these sensor outputs under controlled laboratory conditions.

Future research may wish to compare the use of the DBT CA in the present study, versus a control trial of the warm-up alone. This may be complemented by additional measures such as electromyography activity of upper-limb muscles of interest during the punch tests, to monitor potential changes in neural activity. Further research is required on the use of wearable technology in combat sports. The current authors make no effort to mask the limitations of the sensors used, though we also understand the usefulness that this type of technology can bring, especially as accuracy and reliability of these devices improves with technological advancements. Additionally, individual differences such as strength levels, training age, or body composition were not analyzed in relation to PAPE response. Although all participants had similar training backgrounds and experience levels, it is acknowledged that neuromuscular characteristics and responsiveness to conditioning activities can vary significantly between individuals. Future studies should include sufficient sample sizes and statistical power to explore such individualized response patterns, in line with recent developments in personalized sports training. A final limitation is the lack of individualized analysis of PAPE. While participant characteristics such as age, height, weight, and training experience were recorded, we did not statistically analyze how these factors may have influenced the PAPE response. Given the known variability in neuromuscular adaptations across individuals, such differences in anthropometrics, training history, or technical execution could affect performance outcomes. Future research with larger samples should consider stratified or regression-based analyses to account for individual differences and better understand the personalization of conditioning strategies.

One notable limitation is the absence of a “standardized warm-up only” control group. While this restricts causal claims about the absolute efficacy of the interventions, the study was designed to evaluate the comparative effectiveness of two conditioning activities (DBT vs. DBP) under identical pre-activation conditions. This allowed for a controlled within-subject comparison while avoiding additional participant fatigue and complexity that a third condition might introduce. Future studies may consider including a warm-up only control group to further isolate the independent contribution of CAs.

## 6 Conclusion

To conclude, both the DBT and DBP produced positive effects on punch performance in the present study, though the DBT seemingly produced greater pre-post changes, evidenced by larger mean differences and effect sizes. Consideration to dumbbell load and recovery time, between 5% and 8%5RM of bench press load induce the greatest PAPE effect, and the recovery interval of between 8 and 12-min was considered optimal, though these varied between the rear hand straight and the jab. Future research should focus on an individualized analysis, and also strive to understand the reliability and validity of the wearable technology used in the present study in controlled and uncontrolled scenarios. Lastly, the authors note that future research may wish to compare the DBT against a control trial to fully understand the effect of the CA, compared to a standardized warm-up only. Future research should consider including a no-CA control group to isolate the absolute impact of conditioning activities beyond warm-up alone.

## Data Availability

The original contributions presented in the study are included in the article/supplementary material, further inquiries can be directed to the corresponding author.
